# Safety of solid oncology drugs in older patients: a narrative review

**DOI:** 10.1016/j.esmoop.2024.103965

**Published:** 2024-10-30

**Authors:** A. Rousseau, A. Géraud, R. Geiss, A. Farcet, J.-P. Spano, A.-S. Hamy, P. Gougis

**Affiliations:** 1 Department of Medical Oncology, Pitié-Salpêtrière, Assistance Publique - Hôpitaux de Paris (AP-HP), Paris, France; 2 Department of Medical Oncology, Institut Paoli-Calmette, Marseille, France; 3 Department of Medical Oncology, Institut Curie, Université Paris Cité, Paris, France; 4 Residual Tumor and Response to Treatment, RT2Lab, INSERM, U932 Cancer & Immunity, Institut Curie, Université Paris Sciences Lettres, Paris, France; 5 Sorbonne Université, Institut National de la Santé et de la Recherche Médicale (INSERM), Assistance Publique - Hôpitaux de Paris (AP-HP), Centre d’Investigation Clinique (CIC-1901), Pharmacology Department, Pitié-Salpêtrière Hospital, Paris, France

**Keywords:** *geriatric oncology*, pharmacology, anticancer agents, dose adaptation, elderly population

## Abstract

The older population represents ∼50%-60% of the population of newly diagnosed patients with cancer. Due to physiological and pathological aging and the increased presence of comorbidities and frailty factors, this population is at higher risk of serious toxicity from anticancer drugs and, consequently, often under-treated. Despite the complexity of these treatments, a good knowledge of the pharmacology of anticancer drugs and potentially risky situations can limit the emergence of potentially lethal toxicities in this population. This review focuses on optimizing systemic oncology treatments for older patients, emphasizing the unique characteristics of each therapeutic class and the necessity for a precautionary approach for this vulnerable population.

## Highlights

• Older population represents ∼50%-60% of newly diagnosed cancer patients.
• This population is at higher risk of anticancer drug toxicity due to comorbidities and age-related physiological changes.
• Understanding anticancer pharmacology and risks associated with age can prevent severe toxicities in older patients.

## Graphical abstract

**Figure:**
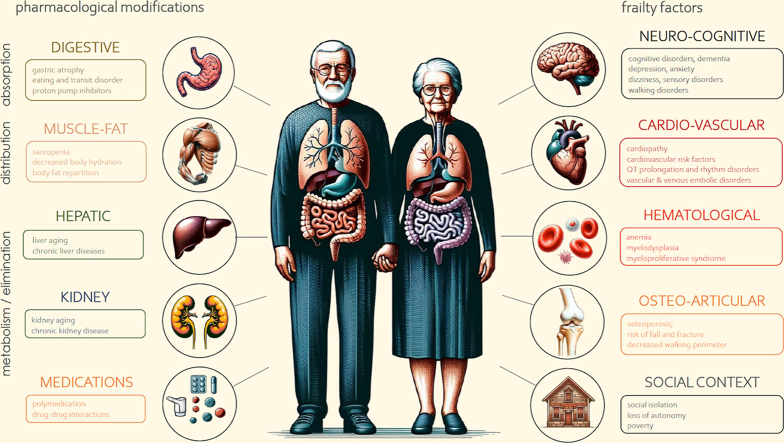

## Introduction

On 4 December 2023, 13 out of 52 (25%) new molecules approved by the European Medicine Agency in 2023 were anticancer drugs.^1^ Most of these new therapies are oral molecular targeted therapies (oMTTs), monoclonal antibodies (mAbs), antibody–drug conjugates (ADCs) or hormonal therapies. However, in 2023, cytotoxic chemotherapies still retained a major role in cancer management, alone or in combination with other therapeutic classes.^2^ Chemotherapies are usually prescribed at a dose at risk of toxicity, and their use in the geriatric population represents a threefold challenge.^3^ Firstly, the clinical data about their tolerance within the older population is often missing since they are frequently excluded from clinical trials.^4^ Older patients (over 65 years old) represent ∼50%-60% of patients with new cancer diagnoses, although they represent only 22%-32% of clinical trial enrollments.^5^, ^6^, ^7^ Secondly, anticancer drugs often have a narrow therapeutic index and have generally been developed at dose levels just below the maximum tolerated dose for populations in good general health.^8^ Thirdly, the geriatric population is particularly fragile, with an increased frequency of toxicities from standard treatment regimens.^9^ With age, individuals experience a progressive decline in the functions of different organs, such as the kidney and the liver, which can lead to substantial changes in the pharmacokinetics and pharmacodynamics of anticancer drugs.^10^ Similarly, the number of comorbidities and comedications also increases significantly with age and can increase the risk of toxicity due to drug–drug interactions (DDIs) or comorbidities.^11^^,^^12^

The aim of this review is to summarize the general principles of anticancer treatments and their adaptation to the geriatric population with a focus on the particularities of classes and main molecules.

## Pharmacological factors of geriatric frailty

### Impact of aging on the pharmacology of anticancer drugs

Physiological aging of the body leads to changes in the various systems involved in drug pharmacokinetics, namely absorption, distribution, metabolism, and excretion.^10^

Absorption of oral drugs could be impaired by mucosal atrophy, reduced intestinal mobility, and reduced splanchnic vascularization.^13^ The absorption of drugs like capecitabine or pazopanib could also be affected by the use of proton pump inhibitors (PPIs) which increase gastric pH and are very frequently prescribed or self-medicated in people with cancer.^14^, ^15^, ^16^

Body composition changes with aging, with an increase in fat mass and a decrease in muscular mass and water content. This could affect the distribution volume of hydrophobic anticancer agents such as oMTTs. Sarcopenia, i.e. the progressive loss of muscle mass and function, can modify drug pharmacokinetics, in particular the volume of distribution, by altering the distribution of drugs in tissues.^17^ It can also change their pharmacodynamics and has emerged as an important factor to predict the toxicity and effectiveness of anticancer drugs.^18^, ^19^, ^20^ Sarcopenia significantly impacts the effective administration of chemotherapy protocols, with increased toxicity and reduced efficacy. This is partially due to weight-based dose adjustments that fail to consider variations in body composition.^21^ The evaluation of body composition using computed tomography scans has been shown to be prognostic in patients undergoing anticancer treatments, including immunotherapy.^22^^,^^23^ This screening could be particularly valuable in the older population.

Drug metabolism and excretion could be affected by renal and hepatic aging causing a risk of toxicity due to excessive drug exposure. Dosage adjustments may be necessary to maintain a similar exposure level to those of a population that would have normal renal or hepatic elimination. There is a decline in cytochrome P450 activity starting at the age of 40 and a reduction of 30% of baseline activity in individuals above 70 years old^24^ for patients with healthy livers. This could be further decreased with hepatic impairment secondary to liver pathologies, and specific scores such as the FIB-4 score could predict anticancer toxicity.^25^

Kidney function naturally declines and nephron loss is estimated at 7.3% per decade.^26^ In oncology, the frequency of older patients with renal failure is often underestimated and could range from 30% to 60%.^27^^,^^28^

Factors associated with geriatric frailty and proposed mitigation methods are summarized in Table 1.

**Table 1:** General principles of frailty factor management for anticancer drug prescription in the geriatric population

Frailty factor	Risk	Risk mitigation	Refs
Renal failure	- Reduced renal excretion of hydrophilic molecules and their metabolites - GFR underestimation with Cockroft formula	- Dosage adjustment for drugs with renal excretions - Standard nephroprotective measures - Use of more suitable formulas: aMDRD, CKD-epi	^27^^,^^56^^,^^113^^,^^114^
Liver failure	- Decreased number of cytochromes - Decreased hepatic clearance	- Dosage reduction	^27^^,^^115^
Sarcopenia	- Increased risk of toxicity - Increased half-life of hydrophilic molecules - Decreased volume of distribution of hydrophobic molecules - Hypoalbuminemia - Marker of chronic inflammation favoring immunosenescence	- Dosage reduction	^18^^,^^116^
Polymedication	- More frequent in older cancer patients than in other older patients - Risk of drug–drug interaction with possible decrease in efficacy or increase in toxicity - Clinically significant interactions mainly mediated by cytochromes P450 and antacids - Self-medication increases the risk of interaction	- Medication reconciliation - Discontinuation of drugs with a limited role - De-prescribing - No self-medication	^11^^,^^32^
Comorbidities	- Comorbidities associated with sub-optimal oncology treatment - Majority of patients have one major comorbidity, and half have more than one - HBP, diabetes, COPD, and heart failure are the most frequent comorbidities (tobacco-related cancers)	- Multidisciplinary geriatric assessment in patients treated for cancer	117, 118, 119
Loss of autonomy	- Disability, social isolation, and neurocognitive impairment can increase the risk of toxicity and reduce the resources available to cope with it	- Geriatric scales for assessing the benefit–risk of treatments	^42^
QT prolongation	- Common with kinase inhibitors (one in three molecules) and endocrine therapies - Increased risk of QT prolongation, rhythm disturbances (Torsades de Pointes), and sudden death in older patients, due to drug-related risks - Supportive care drugs such as ondansetron or methadone also a source of risk	- Regular measurement of QTc before/after administration of high-risk molecules	120, 121, 122, 123, 124

aMDRD, abbreviated MDRD; CKD-epi, chronic kidney disease epidemiology; COPD, chronic obstructive pulmonary disease; GFR, glomerular filtration rate; HBP, high blood pressure; MDRD, modification of diet in renal disease; QTc, QT corrected.

### Polymedication and drug interactions

Age is accompanied by an increase in the number of comorbidities and chronic treatments outside of cancer.^29^^,^^30^ Polypharmacy is defined as taking at least five medications. Polymedication is particularly common among older cancer patients,^31^ and it is associated with an increase in mortality in the older population^12^ that could be mediated by DDI. More generally, the potential for DDIs in these patients can lead to decreased efficacy or increased toxicity, which can be mitigated through the discontinuation of medications that offer limited therapeutic value, necessitating careful medication reconciliation to manage the increased risk.^11^^,^^32^

The majority of drug interactions with a clinically significant impact are mediated by cytochromes and by changes in drug absorption due to antacids and PPIs.^16^^,^^17^ When the drug’s active molecule is subject to interactions, this can lead to underexposure, which may reduce its efficacy, or overexposure, which increases toxicity.

Indeed, the vast majority of oral molecularly targeted therapies (>90%), taxanes, vinca-alkaloids, as well as certain endocrine therapies (anti-androgens) are metabolized by CYP3A4.^17^ Particular vigilance is therefore required for drugs and herbal therapies that strongly interact with this cytochrome and should be avoided or treated with caution with most of these prescriptions. The most frequently prescribed CYP3A4 inhibitors are azole antifungal agents (voriconazole, pasaconazole, fluconazole), antivirals (ritonavir, saquinavir), and herbal remedies (grapefruit, Seville orange, goldenseal)^33^ among others. CYP3A4 inducers include, but are not limited to, antiepileptics (carbamazepine, phenytoin, phenobarbitals), rifampicin, ritonavir and St. John’s wort.^33^ Certain drugs, with multiple and complex interactions, may require drug conciliation or systematic use of interaction detection software.^34^

### Dose adaptation and geriatric assessment in patients treated for cancer

**Figure 1:**
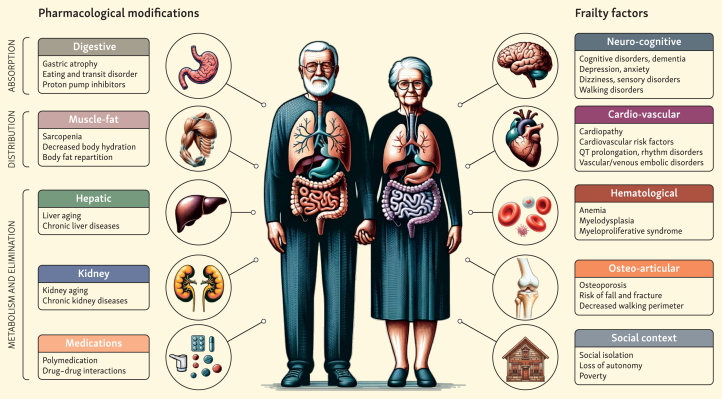
Factors associated with geriatric frailty and risk of toxicity from anticancer drugs.

Geriatric assessment in patients treated for cancer is a multidisciplinary evaluation that could involve a wide range of health professionals (doctors, nutritionists, social workers, psychologists, pharmacists) to evaluate patients with high risk of toxicity^35^ from a variety of sources of frailties in this population (comorbidities, cognitive disorder, depressive syndrome, risk of falls, polymedication, malnutrition, and social isolation),^35^ summarized in Figure 1. These oncogeriatric frailties could be associated with a higher risk of severe toxicity (grade ≥3) or mortality.^36^^,^^37^

The management of correctable weaknesses is the first step, such as the establishment of nutritional support in the case of malnutrition or psychiatric and psychological evaluation in the case of depressive syndrome. When the geriatric assessments favor the initiation of systemic treatment, dose adaptation and strengthening of supportive care must be discussed. However, when the risk of toxicity is too high in severely impaired patients, particularly with tumors of poor prognosis, exclusive supportive care may also be considered.

To assist the clinician in carrying out this complex assessment, scores that include pharmacological, cancer, and geriatric variables were developed to help predict the risk of severe toxicity and hospitalization. The CRASH score (Chemotherapy Risk Assessment Scale for High-Age Patients) and the CARG score (Cancer and Aging Research Group) are two scores that predict the risk of toxicity more accurately than the World Health Organization (WHO) performance status.^38^, ^39^, ^40^ However, in a prospective external cohort of 248 patients, these two scores were unable to predict chemotherapy-induced severe adverse events (AEs).^41^ On the other hand, factors such as cancer type, performance status, comorbidities, and body mass index were independently associated with grade 3 or higher toxicities. Additionally, these scores were not designed to predict outcomes for molecular targeted therapies or immunotherapies, limiting their overall relevance. Therefore, treatment decisions should not rely solely on these two scores when considering cancer therapy.

The GAP70+ study evaluated the contribution of geriatric assessment-based interventions compared with usual care, and demonstrated a 20% reduction in severe toxicities without a reduction in overall survival in older patients with advanced solid or hematological cancer.^42^ The GAIN study, which included 605 patients, showed similar benefit of a multidisciplinary geriatric assessment-driven intervention to limit chemotherapy-adverse effects at treatment initiation.^43^ Another study evaluated the impact of reducing the dose of bi-chemotherapy (oxaliplatin and capecitabine) by −20% and −40%, respectively, in older, frail patients with high-grade digestive cancer.^44^ There was a significant reduction of the risk of toxicity without compromising tumor control (non-inferiority demonstrated in progression-free survival and overall survival).

### Heterogeneity of older population

Aging is associated with a range of physiological changes that can vary significantly between individuals.^45^ These changes have a substantial impact on the pharmacokinetics and pharmacodynamics of drugs, potentially influencing different medications based on their biophysical characteristics and target properties.^46^ For instance, variations in body composition, such as increased fat mass and decreased lean muscle mass, affect the volume of distribution of lipophilic and hydrophilic drugs in distinct ways (Figure 1).^47^ Similarly, age-related changes in liver function, including decreased hepatic blood flow and enzyme activity, can significantly affect drug metabolism, particularly for drugs processed via the cytochrome P450 system.^48^ Renal function, which often declines with age, directly impacts the excretion of many medications and their metabolites, necessitating dose adjustments based on renal clearance. Functional and cognitive capacities also vary greatly among older adults, further contributing to the heterogeneity of the geriatric population.^49^ Functional status, which include the ability to carry out activities of daily living (ADLs) and instrumental ADLs, can influence a patient’s ability to adhere to complex medication regimens. Cognitive impairment, ranging from mild cognitive decline to dementia, affects the ability to understand and follow medication instructions, increasing the risk of errors and adverse outcomes. Consequently, older patients require more comprehensive clinical assessments and individualized treatment approaches, as the ‘one size fits all’ model, while less harmful in younger populations, poses greater risks of adverse effects in the older population.

### Cardiotoxicity of anticancer drugs

Cardiovascular risk factors, diseases, and complications increase significantly with age^50^, ^51^, ^52^ and can complicate the administration of molecules with known cardiovascular complications such as anthracyclines (doxorubicin, epirubicin), anti-human epidermal growth factor receptor 2 (HER2) agents (trastuzumab, pertuzumab, trastuzumab-emtansine, trastuzumab deruxtecan), and antiangiogenic agents (bevacizumab, sunitinib).^53^ Patients aged 80 and above are at high risk for cardiovascular toxicity when treated with anthracyclines and HER2-targeted therapies, while vascular endothelial growth factor inhibitors, breakpoint cluster region-Abelson inhibitors, and multiple myeloma therapies pose high risks in those over 75.^53^ As a result, patients over 80 receiving anthracycline treatment should undergo a thorough baseline cardiovascular evaluation, with troponin and brain natriuretic peptide assessments before each injection, and a transthoracic echocardiography every two cycles.

This population is also at greater risk of rhythmic complications such as atrial fibrillation (abiraterone, ibrutinib) or QT prolongation and Torsades de Pointes (ribociclib, sunitinib, pazopanib, osimertinib, etc.)^54^ for which dedicated electrocardiogram (ECG) and/or cardiovascular monitoring can enable early detection, preventing them and limiting their consequences.

The impact of age on 5-fluorouracil coronary vasospasm remains debated, and bolus administration significantly increases it. Older patients may have a reduced risk of vasospasm.^55^^,^^56^ Risk mitigation strategies include limiting bolus administration, with no other routine adaptations currently recommended. Oral antiangiogenics (e.g. cabozantinib, axitinib, sunitinib, lenvatinib, pazopanib) have an increased likelihood of AEs in older adults, despite clearance not being directly linked to age. QT prolongation, Torsades de Pointes, and sudden death are additional risks. Mitigation includes starting treatment at lower doses, close cardiovascular monitoring for comorbid patients, and comprehensive medication reconciliation.^57^, ^58^, ^59^, ^60^ For ibrutinib, AEs such as atrial fibrillation are more common in older patients, although toxicity is not dose dependent. While no dosage adaptations are recommended, close cardiovascular monitoring is essential for those with cardiovascular comorbidities, and includes baseline cardiovascular transthoracic echocardiography and regular blood pressure monitoring.^53^ Similarly, trastuzumab cardiovascular toxicity is not dose dependent, although this risk is slightly higher in the geriatric population. It manifests as a left ventricular ejection fraction (LVEF) decline, particularly in those with pre-existing cardiac comorbidities. To mitigate these risks, no specific dosage adjustments are recommended, but rigorous cardiovascular monitoring, which could include troponin and brain natriuretic peptide dosage every cycle, and transthoracic echocardiography every 3 months might limit cardiac complications.^13^^,^^53^^,^^61^^,^^62^

Several other cancer treatments can also lead to clinically significant cardiovascular events. Agents such as cyclophosphamide, cisplatin, ifosfamide, and taxanes (paclitaxel and docetaxel) have been associated with myocardial dysfunction and heart failure, though these occurrences are rare, and no specific guidelines exist for managing them in older patients.^53^

### Drug compliance

Among older adults, non-compliance with prescribed medication regimens is a common issue^63^ that can lead to suboptimal management of chronic diseases, increased hospitalizations, and higher mortality rates. Understanding the multifaceted nature of drug compliance in older patients is essential for developing effective strategies to improve adherence.^64^ Patients with dementia may forget to take their medications, take incorrect dosages, or take medications at the wrong times, leading to poor therapeutic outcomes. Depression and anxiety, which are prevalent among older adults, can negatively impact medication adherence. Lower health literacy, which is more prevalent in older populations, can lead to misunderstandings about the purpose, dosage, and timing of medications, further compromising adherence. The complexity of medication regimens is another critical determinant of compliance in older adults. Complex regimens with multiple daily doses, varying instructions, and frequent changes increase the likelihood of errors and non-adherence.^65^ To mitigate this risk, the International Society of Geriatric Oncology (SIOG) taskforce^65^ recommends that adherence to oral cancer therapy in older adults be managed through proactive, multidisciplinary approaches tailored to individual patient needs and available resources. They emphasize the importance of selecting suitable patients for oral therapy and implementing ongoing monitoring interventions to optimize adherence and improve treatment outcomes. Involving various health care professionals, such as clinical pharmacists, in the care of older adults with dementia can further enhance medication adherence and reduce the risks of non-compliance.^66^

## Cytotoxic agents in the geriatric population

### General considerations

Unlike other drug classes (oMTTs or mAbs), which share similar pharmacological characteristics, the pharmacology of cytotoxics is highly varied. Of note, all spindle poisons (taxanes and vinca-alkaloids) are metabolized in the liver via CYP3A4 mainly (docetaxel, vinorelbine) or partially (paclitaxel), and adaptation of these drugs in patients with renal insufficiency is generally not needed or is moderate.^27^^,^^56^ Considerations and risk mitigations for main cytotoxic anticancers are summarized in Table 2. In selected patients with advanced gastrointestinal cancers and normal independent ADL, polychemotherapy can be safely used,^67^ though it may result in higher rates of dose reductions or interruptions.^68^ However, in other cancer types, such as metastatic breast cancer, scientific societies recommend single-agent chemotherapy over polychemotherapy.^69^

**Table 2:** Therapeutic attitudes adapted to older patients

Anticancer drug	Risk in the geriatric population, at-risk populations	Therapeutic attitudes in the older and at-risk subpopulations	Refs
**Chemotherapy**			
Doxorubicin	- High blood pressure and age increase the risk of cardiotoxicity - Risk of non-cardiac AE increased but not directly related to age - Hypoalbuminemia/sarcopenia is a risk factor for overdose - Increased toxicity in cases of hepatic dysfunction	- No systematic adaptation in older patients - Adaptation to be considered in malnourished patients - Adaptation or contraindication if hepatic dysfunction	^116^^,^^125^, ^126^, ^127^
Cyclophosphamide	- Reduced plasma clearance in cases of low body weight, hypoalbuminemia, and renal impairment (active metabolites) - Potential drug interactions via CYP3A4	- Start at reduced dose only if significant comorbidities - 20%-30% reduction if renal insufficiency	^116^^,^^128^
Docetaxel	- Risk of AEs, particularly infectious, increased without being directly age-related - Drug interactions via CYP3A4 - Reduced elimination if hepatobiliary function impaired - Asian population seems more vulnerable to toxicities	- Regimen 50 mg/m^2^ every 2 weeks in prostate cancer - Adaptation or contraindication if hepatic dysfunction	^116^^,^^129^^,^^130^
Cabazitaxel	- Drug interactions via CYP3A4 - Higher risk of febrile neutropenia	- Prevention of neutropenia by G-CSF for patients >75 years old - Regimen 16 mg/m^2^ every 2 weeks seems to be less toxic	^131^^,^^132^
Paclitaxel	- Hypoalbuminemia is a risk factor of overdose - Drug interactions via CYP3A4 (and CYP2C8) - Reduced elimination if hepatobiliary function is impaired	- No systematic adaptation - Weekly schedule is less hematologically toxic - Dose reduction or no administration if hepatic dysfunction	^116^^,^^133^
Carboplatin	- Hematological toxicity dependent on the dose, itself very dependent on renal function - Malnourished patients: may falsely present normal renal function	- Vigilance in renal insufficiency and adaptation of the AUC according to GFR estimation - Prevention of neutropenia by G-CSF in the slightest doubt about the patient's fragility	^116^
Cisplatin	- Age is an independent factor of poorer renal elimination - Older subjects present more toxicities, in particular neurotoxicity, ototoxicity, and nephrotoxicity - Hyperhydration may be poorly tolerated (acute heart failure)	- Administration only in very selected patients, at reduced dose and at a slow delivery - Prefer alternatives (carboplatin, oxaliplatin) if possible	^114^^,^^116^^,^^134^
Oxaliplatin	- Reduced clearance in patients with renal insufficiency - No association between age and toxicity, particularly hematological and neurological	- No adaptation recommended routinely - Dose reduction if severe renal failure	^116^^,^^135^
5-fluorouracil	- Data on the role of age as an independent factor of toxicity are contradictory - If administered as a bolus, toxicity is increased, particularly hematological - Reduced risk of vasospasm in older patients	- Limit bolus administration - No other adaptations recommended routinely	^55^^,^^116^
Capecitabine	- Less hand–foot syndrome and hematotoxicity compared to 5-FU i.v. - Increased AEs in renal failure patients - Drug interaction with antacids	- Scheme at 1000 mg/m^2^ x 2/day better tolerated and effective than standard dosages - Dosage reduction in patients with renal insufficiency - Adaptation of antacid treatments (PPI relay for antihistamine)	^116^^,^^136^
**Immunotherapies**			
Anti-PD-1/PD-L1	- Similar toxicity profiles in the geriatric population	- No adaptation recommended	^137^^,^^138^
Ipilimumab	- Similar toxicity profiles in the geriatric population	- No adaptation recommended	^139^^,^^140^
**Targeted therapies**			
Cetuximab	- Similar toxicity profiles in the geriatric population	- No adaptation recommended	^141^
Trastuzumab	- Risk of decrease in LVEF slightly increased in the geriatric population, and during pre-existing cardiac comorbidities, but not dose dependent	- No adaptation recommended - Rigorous cardiovascular monitoring during treatment	^13^^,^^61^^,^^62^
Rituximab	- More frequent severe AEs, particularly cardiac (supraventricular arrhythmia) and infectious (pneumonitis), uncertain relationship with dose for the treatment of hematological malignancies	- No adaptation recommended	^142^^,^^143^
Bevacizumab	- More frequent AEs (hypertension, venous and arterial thromboembolism, proteinuria, fistulas)	- No adaptation recommended - Assessment of benefit/risk which may be disadvantageous in patients with significant cardiovascular risk	144, 145, 146
Oral antiangiogenics (cabozantinib, axitinib, sunitinib, lenvatinib, pazopanib)	- Drug interactions via CYP3A4 (all) and antacids (pazopanib) - Strong inter-individual variability - Clearance not directly linked to age but AEs are more frequent - QT prolongation, TdP (pazopanib, vandetanib), and sudden death (sunitinib) - Be careful of liver failure, particularly for the most hepatotoxic (cabozantinib, pazopanib)	- Start at a lower dose level - Close cardiovascular monitoring if cardiovascular comorbidities - Medication reconciliation	57, 58, 59, 60
Osimertinib	- Risk of AEs increased without being directly linked to age - Interactions via CYP3A4 - QT prolongation and TdP	- No adaptation recommended - ECG before (and after) initiation	147, 148, 149
Ibrutinib	- AEs more common in older patients, notably AF, but non-dose-dependent toxicity - Drug interactions via CYP3A4	- No adaptation recommended - Close cardiovascular monitoring in patients with cardiovascular comorbidity	150, 151, 152
Imatinib	- AEs (edema) more common in older subjects - Drug interactions via CYP3A4 (as substrate) and via CYP2D6 (inhibitor)	- Consider dose reduction in fragile patients, and depending on the pathology - Medication reconciliation	153, 154, 155
Olaparib	- AEs do not seem to be modified by age - Drug interactions via CYP3A4 - Elevations in serum creatinine without impairment of renal function	- No adaptation recommended - Evaluate renal function with cystatin C if serum creatinine increases	156, 157, 158, 159
CDK4/6 inhibitors (palbociclib, ribociclib, abemaciclib)	- AEs appear similar in older patients - Drug interactions via CYP3A4 - QT prolongation and TdP with ribociclib	- No adaptation recommended - Vigilance on diarrhea with abemaciclib - ECG monitoring for ribociclib	160, 161, 162, 163
**Endocrine therapies**			
LHRH agonists (goserelin, leuprorelin, triptorelin) LH antagonists (degarelix*)*	Cardiac toxicities could be higher in LH agonists	- No adaptation recommended	109, 110, 111
Abiraterone	- Cardiac AEs and edema slightly more common in older subjects, but toxicity does not seem dose dependent - Drug interactions via CYP3A4 (substrate) and CYP2D6 (inhibitor)	- No adaptation recommended	164, 165, 166
Anti-androgen (enzalutamide, darolutamide, apalutamide)	- More frequent fall rates and neurocognitive AEs - Drug interactions with inh/ind CYP3A4 (enzalutamide, darolutamide) and CYP2C8 (enzalutamide). May modify the PK of other drugs (CYP2C9, CYP2C19) - QT prolongation and TdP	- No adaptation recommended - Darolutamide to be preferred if there is a risk of epilepsy - Medication reconciliation - ECG monitoring if cardiovascular comorbidities	167, 168, 169, 170, 171
Aromatase inhibitors (anastrozole, letrozole, exemestane)	- Similar AEs in older patients	- No adaptation recommended	^172^^,^^173^
Fulvestrant	- Similar AEs in older patients	- No adaptation recommended	^104^

Data adapted and synthesized from summaries of product characteristics from ANSM (https://base-donnees-publique.medicaments.gouv.fr/), the European Medicines Agency (https://www.ema.europa.eu/en/medicines), the US Food and Drug Administration (https://www.accessdata.fda.gov/scripts/cder/daf/index.cfm), and the National Institutes of Health (NIH) prescription website, DailyMed® (https://dailymed.nlm.nih.gov/dailymed/).

5-FU, 5-fluorouracil; AEs, adverse events; AF, atrial fibrillation; AUC, area under the curve; CDK, cyclin-dependent kinase; CYP, cytochrome P450; ECG, electrocardiogram; G-CSF, granulocyte colony-stimulating factor; GFR, glomerular filtration rate; LHRH, luteinizing hormone-releasing hormone; LVEF, left ventricular ejection fraction; PD-1, programmed cell death protein 1; PD-L1, programmed death-ligand 1; PK, pharmacokinetics; PPI, proton pump inhibitors; TdP, Torsades de Pointes.

Patients fit enough to be included in early phase trials exhibit comparable safety and efficacy outcomes.^70^

### Management and prevention of febrile neutropenia

Febrile neutropenia (NF) is a potentially serious complication that can occur frequently with certain chemotherapy protocols. The risk of mortality from NF increases significantly with the number of comorbidities and can reach over 20% in cases of high comorbidity with the administration of neutropenic molecules. The European Society of Medical Oncology recommends primary prophylaxis with granulocyte colony-stimulating factor (G-CSF) in cases of NF risk of over 20%, and in cases of NF risk between 10% and 20% associated with comorbidities or age over 65.^71^ The use of primary prophylaxis can significantly reduce the risk of NF and hospitalization in older patients receiving chemotherapy at high or intermediate risk of NF.^71^ However, the risk of NF is often underestimated in the older population with combinations combining anthracyclines or carboplatin, as well as for certain monotherapies such as docetaxel.^72^ Given the risk of death associated with this avoidable complication, prescribing G-CSF should be readily considered in this population. Similarly, when NF is detected, even in the absence of an infectious warning sign, hospital care should be rapidly initiated, with bacteriological sampling and initiation of broad-spectrum antibiotics.

## Monoclonal antibodies and antibody–drug conjugates

### Monoclonal antibodies

mAbs are eliminated by endocytosis and proteolysis in the vascular endothelium.^73^ Thus, patients with renal or hepatic insufficiency have no major changes in their exposure and they are not subject to cytochrome-mediated drug interactions. Due to the absence of human ether-à-go-go-related gene inhibition and their specificity, they are not at risk of QT prolongation.^54^ They also have a broader therapeutic index than cytotoxic agents and their toxicities are often not dose dependent.^74^ Although data on the pharmacokinetics of these drugs in geriatric populations are lacking, few differences are expected in this population. As a result, initial dosage adjustments are rarely necessary when prescribing these drugs.

On the other hand, the geriatric population, often burdened with comorbidities like cardiovascular disease, is more vulnerable to organ-specific toxicities. As a result, these patients may require closer monitoring and specialized pre-treatment assessments to mitigate risks. As an illustration, bevacizumab increases hypertension and thromboembolic risk and may require prior cardiovascular assessment, with a multidisciplinary evaluation of the benefit–risk ratio.^75^ Cardiovascular monitoring can also help anticipate and limit the occurrence of serious cardiovascular complications.^53^ Antibodies targeting epidermal growth factor receptor (EGFR) can be safely used in fit older patients, but in vulnerable patients, their dose should be adapted to avoid toxicity.^76^

Regarding immune checkpoint inhibitors (ICIs), numerous studies have found no significant difference in the risk of toxicity in older patients with either anti-cytotoxic T-lymphocyte associated protein 4 (cytotoxic T-lymphocyte antigen 4) or anti-programmed cell death protein 1/programmed death-ligand 1 (PD-1) immunotherapies or the combination of the two for most toxicities.^77^ But other studies found conflicting results with increased risk of myocarditis and myositis.^78^^,^^79^ ICI-induced myocarditis-myositis increased risk in the older population could be linked to thymus involvement, which is a gatekeeper preventing this toxicity.^80^ Troponin should be monitored in this population, particularly in the 3 first months during which most of the ICI-induced cardiotoxicity occurs.^53^^,^^79^ The benefit of prescribing dual anti-PD-1/anti-cytotoxic T-lymphocyte antigen 4 therapy must also take into account the significant risk of developing grade ≥3 toxicity with this combination, which may have greater consequences in a patient with loss of autonomy or impaired neurocognitive capacities.^81^, ^82^, ^83^ In a pharmacovigilance study, age over 65 was a risk factor of death with immune-related AEs after adjustment on other variables (odds ratio = 1.16, 95% confidence interval, 1.1-1.2).^79^

Similar caution should be exerted when using these ICIs (nivolumab, pembrolizumab) in combination with oMTTs (axitinib, lenvatinib, cabozantinib), as in the management of advanced kidney cancer.^84^, ^85^, ^86^

### Antibody–drug conjugates

ADCs are a novel class of anticancer agents that combine mAbs with potent cytotoxic drugs, offering targeted therapy with potentially improved efficacy and safety profiles. In older patients, pharmacokinetic and pharmacodynamic changes, along with comorbidities, necessitate careful consideration when using these agents.

Trastuzumab deruxtecan (T-DXd) is an anti-HER2 ADC linked to a topoisomerase I inhibitor (DXd), approved for various HER2-positive and HER2-low metastatic cancers. Clinical trials have demonstrated that T-DXd maintains similar efficacy in patients aged ≥65 years compared to younger patients.^87^ However, higher rates of AEs were observed in older patients treated with T-DXd compared to younger patients.^87^ Serious AEs occurred in 32.2% of older patients versus 24.3% in those under 65 years. Grade 3 or higher treatment-emergent adverse events (TEAEs) were more frequent in the older group (65.5% versus 53.6%) and discontinuation of T-DXd due to TEAEs was also higher (25.4% versus 18.7%). The incidence of any-grade drug-related interstitial lung disease/pneumonitis (17.5% versus 11.8%) and that of fatal pneumonitis were also higher (0.9% versus 0.6%). Given the increased risk of pulmonary toxicity, particularly in those with pulmonary comorbidities such as smoking history, lung disease, prior interstitial lung disease, renal insufficiency, or previous thoracic radiotherapy, close monitoring for respiratory symptoms is crucial in older patients receiving T-DXd.

Sacituzumab govitecan is an anti-Trop-2 ADC delivering SN-38, the active metabolite of irinotecan, approved for metastatic breast cancer and urothelial carcinoma.^88^, ^89^, ^90^ While efficacy in patients aged ≥65 years is comparable to that in younger patients, older individuals may experience higher rates of AEs leading to dose reductions.^91^ Management strategies for older patients include proactive monitoring of blood counts, early initiation of colony-stimulating factors to mitigate neutropenia, and prompt treatment of diarrhea with antidiarrheal agents to prevent dehydration and renal impairment. In patients aged 65 years and older with metastatic triple-negative breast cancer, sacituzumab govitecan demonstrated similar efficacy compared to younger patients. However, the safety profile differed across age groups, and a higher incidence of TEAEs leading to dose reductions was observed in patients aged ≥65 years compared to those under 65 years (33% versus 19%).^91^ In metastatic urothelial carcinoma, the TROPHY-U-01 study reported a median patient age of 65 years, with 27% of patients over 75 years old. The most common grade ≥3 TEAEs were neutropenia (35%), anemia (14%), diarrhea (10%), and febrile neutropenia (10%). A significant proportion of patients (30.1%) required the use of G-CSF, including 18% during the first cycle of treatment. For older patients, particularly those at high risk of dehydration, it is crucial to promptly assess hydration status and renal function in the event of diarrhea and to initiate symptomatic treatment with antidiarrheal agents early. Altogether, this indicates that older patients may require closer monitoring and potential dose adjustments to manage toxicity effectively and/or manage symptoms.

Enfortumab vedotin is an ADC targeting Nectin-4 and linked to monomethyl auristatin E (MMAE), approved for the treatment of metastatic urothelial carcinoma as monotherapy or in combination with pembrolizumab.^92^ In clinical trials involving cisplatin-ineligible patients (creatinine clearance <60 ml/min) with locally advanced or metastatic urothelial carcinoma, the median age was 75 years, and the objective response rate was 52%.^92^ Common treatment-related adverse events (TRAEs) included alopecia (51%), peripheral sensory neuropathy (47%), and fatigue (34%). Grade ≥3 TRAEs included rash (6%), peripheral neuropathy (8%), and hyperglycemia (6%).^92^ This suggests that enfortumab vedotin is effective in older patients but may pose an increased risk of certain toxicities such as peripheral neuropathy, skin reactions, and hyperglycemia. Close monitoring for these AEs is essential, and dose adjustments or supportive care measures may be necessary to maintain treatment tolerability in this population.

However, data remain limited, and further research is needed,^93^ as clinical trials often lack representativeness of the general population, with new drugs typically being tested in younger patients.^94^

## Oral molecular targeted therapies

oMTTs are a drug class with a wide variety of therapeutic targets, and their therapeutic index is sometimes narrow and similar to that of cytotoxic toxicities, as for oral antiangiogenic drugs.^17^ Some drugs, on the other hand, are better tolerated at effective doses, such as osimertinib, a third-generation EGFR inhibitor.

### Oral antiangiogenic agents

Oral antiangiogenic agents represent a particularly important class in certain cancers where angiogenesis plays a predominant role, such as kidney, liver, sarcoma, and thyroid cancers.^75^ Sunitinib, for example, can be prescribed at a lower dose level (37.5 mg daily dose 4 weeks out of 6 instead of 50 mg daily) in the geriatric population to limit the risk of toxicity, which is dose dependent in this class of drugs.^57^ As these drugs are hepatically metabolized, renal failure has little effect on their pharmacokinetics compared with the normorenal population. However, renal insufficiency can favor the emergence of cardiovascular toxicities, such as hypertension, and dosage adjustment can help limit the risk of their occurrence.

The French PreToxE cohort evaluated the safety profile of oMTTs in patients aged 70 and over. The main class evaluated were antiangiogenic agents and mTOR inhibitors. About 40% of patients experienced clinically significant toxicities, half of which were grade 3 or higher. This led to treatment discontinuation for a third of patients. Risk factors for toxicity were polymedication (≥3 drugs) and antiangiogenic class.^95^

In particularly fragile patients with multiple cardiovascular histories, and for tumors with multiple validated antiangiogenic options, as in clear-cell kidney cancer, it may be preferable to favor drugs with very short half-lives. With these molecules, most adverse effects are rapidly resolved at discontinuation. Axitinib, for example, has a half-life of between 3 and 6 h, and adverse effects are rapidly resolved on discontinuation, compared with drugs with a long half-life such as cabozantinib, which has a half-life of 5 days.^75^ However, this strategy does not mitigate the risk of thromboembolic events, particularly in cases of severe cardiovascular history. When there is a history of poorly controlled arterial hypertension, close cardiovascular monitoring may therefore be necessary.

### Cyclin-dependent kinase inhibitors

Secondary analyses of MONARCH 2 and 3 revealed that abemaciclib resulted in a higher incidence of clinically relevant diarrhea in older patients, along with moderately elevated rates of nausea, decreased appetite, and venous thromboembolic events. However, the rates of neutropenia remained consistent between older and younger patients.^96^ Palbociclib may be better tolerated than abemaciclib, as serious AEs are not more frequent in patients over 80 years old compared to those under 80 years old.^97^ Ribociclib, a potent CYP3A4 inhibitor,^98^^,^^99^ requires particular caution when used in combination with other CYP3A4 substrates, such as statins, due to the risk of statin-increased exposure and rhabdomyolysis.^100^ Furthermore, its QT-prolonging effects can be exacerbated by concomitant use of torsadogenic agents, such as neuroleptics or antidepressants, increasing the likelihood of Torsade de Pointes and, in severe cases, potentially resulting in sudden death.^54^

### Other oral molecular targeted therapies

The variability of tolerability of molecular therapy targets makes generalization difficult, and can vary widely, even within the same class. ALK inhibitors such as alectinib, have a much better safety profile than lorlatinib, with a lower rate of grade 3-4 adverse reactions and treatment discontinuation.^101^^,^^102^ Their metabolism is almost systematically hepatic via CYP3A4 and their pharmacokinetics are, therefore, little affected by renal insufficiency. oMTTs are also commonly associated with QT prolongation, sometimes as a class effect (BRAF inhibitors), or independently of their class, like for ribociclib.^54^

## Endocrine therapies

### Aromatase inhibitors

Aromatase inhibitors (anastrozole, letrozole, exemestane) have a broader therapeutic index than cytotoxics.^103^ Their tolerance is mainly marked by arthralgia, the frequency and severity of which varies widely in the population. In cases where tolerance compromises compliance, it may be considered to switch to a different class of endocrine therapy rather than decrease the dose. Other AEs are dyslipidemia and bone demineralization due to estrogen deprivation. These should be assessed at initiation and throughout the course of treatment (vitamin and calcium supplementation, measurement of bone mineral density, lipid profile, and management of cardiovascular risk factors).^103^ Tamoxifen, a selective estrogen receptor modulator, can be an alternative to aromatase inhibitors in cases of poor tolerance or contraindication (osteoporosis).^103^ However, it is associated with a higher embolic risk and may increase the risk of endometrial cancer through its pro-estrogenic effect.^103^

### Selective estrogen receptor degrader (SERD)

Fulvestrant is metabolized by biotransformation pathways analogous to those of endogenous steroids. Aging has no impact on fulvestrant pharmacology and pharmacodynamics. No dose adaptation is required.^104^

## Inhibitors of androgen receptor pathways

The treatment of prostate cancer is based on androgen deprivation and inhibition of the testosterone/androgen receptor axis [androgen receptor axis inhibitors (ARPI)].

### Abiraterone acetate

Abiraterone acetate inhibits testosterone synthesis through inhibition of cytochrome P450 17A1.^105^ To limit the resulting glucocorticoid deficiency, prednisone is co-prescribed, at a dose of 5 mg for hormone-naïve cancers or 10 mg when there is castration resistance. Prednisone overactivates the mineralocorticoid axis, increasing the risk of hypertension and atrial fibrillation.^54^ Older patients are more sensitive to these risks, however, no dose adjustment is necessary. Regular monitoring of blood pressure, heart rate and rhythm should be systematic.

### Androgen receptor inhibitors

Enzalutamide, apalutamide, and darolutamide are second-generation androgen receptor inhibitors with complex metabolism and elimination. Darolutamide is mainly eliminated by the kidney (63.4% for darolutamide). Its prescription needs to be adapted in cases of severe renal impairment (estimated glomerular filtration rate <30 ml/min/1.73 m^2^). Enzalutamide and apalutamide have a low rate of unchanged elimination through the kidney. The risk of exposure variations due to kidney failure is therefore unlikely, but clinical data are missing in cases of severe renal impairment.^106^^,^^107^

Enzalutamide and apalutamide are strong cytochrome inducers, notably the CYP3A4, and could therefore decrease the exposure of numerous classes of drugs, such as benzodiazepines.^108^ There is, therefore, a high risk of drug interaction with these molecules, and DDI should be verified with both molecules. Darolutamide, on the other hand, has a very low risk of DDI because it is only a weak inducer of CYP3A4.

Finally, testosterone is a QT-shortening hormone, which explains why men generally have a shorter QT than women. Drugs that interact with testosterone secretion or binding are, therefore, often responsible for QT prolongation, including gonadotropin-releasing hormone agonists/antagonists. This risk is particularly pronounced with enzalutamide.^54^ In older patients with cardiovascular comorbidities, it is therefore advisable to prescribe an ECG with measurement of corrected QT (according to Fridericia's formula) before initiating these treatments.

### Agonist and antagonist of LHRH

Agonists (goserelin, leuprorelin, triptorelin) and antagonists of luteinizing hormone-releasing hormone (degarelix) are part of the basic treatment for prostate cancer in situations of hormonal sensitivity or resistance.^109^ Antagonists appear to be associated with a lower risk of cardiovascular toxicities and should be preferred in patients with high cardiovascular risk.^110^^,^^111^

## Conclusion

Despite the complexity of these treatments, a good knowledge of the pharmacology of anticancer drugs and of potentially risky situations can limit the risk of emergence of potentially lethal toxicities in older patients. There is no general pattern for pharmacological considerations with cytotoxic drugs, apart from their narrow therapeutic index, and some molecules require systematic adaptation in all older patients or according to individual frailties. On the other hand, targeted molecular therapies, such as mAbs, are less likely to require dose adaptation for older subjects. The management of oral molecular targeted therapies is mostly class dependent, with antiangiogenic agents often requiring special adaptations or precautions. In contrast, therapies such as osimertinib can be prescribed at usual doses with very good tolerability. Finally, aromatase inhibitors, which are still widely prescribed, do not require adaptation, whereas therapies targeting the androgen receptor axes may prolong the QT or be easily subject to drug interactions. Overall, the ‘*primum non nocere*’ principle is particularly well suited to oncogeriatrics. Systemic treatments, whose prescription is often motivated by interesting results in randomized trials, can ultimately lead to toxic deaths in this population, for which hindsight is often lacking and which is poorly evaluated. An intra-patient dose-escalation approach, starting with a low dose and gradually increasing over the course of cycles, is an approach that deserves to be developed. This could help limit toxicities while maintaining a good dose density.^112^ Complex situations should also encourage joint management with organ specialists.
